# A Paradox of Syntactic Priming: Why Response Tendencies Show Priming for Passives, and Response Latencies Show Priming for Actives

**DOI:** 10.1371/journal.pone.0024209

**Published:** 2011-10-11

**Authors:** Katrien Segaert, Laura Menenti, Kirsten Weber, Peter Hagoort

**Affiliations:** 1 Max Planck Institute for Psycholinguistics, Nijmegen, The Netherlands; 2 Donders Institute for Brain, Cognition and Behaviour, Centre for Cognitive Neuroimaging, Radboud University Nijmegen, Nijmegen, The Netherlands; 3 Institute for Neuroscience and Psychology, University of Glasgow, Glasgow, Scotland, United Kingdom; University of Edinburgh, United Kingdom

## Abstract

Speakers tend to repeat syntactic structures across sentences, a phenomenon called syntactic priming. Although it has been suggested that repeating syntactic structures should result in speeded responses, previous research has focused on effects in response tendencies. We investigated syntactic priming effects simultaneously in response tendencies and response latencies for active and passive transitive sentences in a picture description task. In Experiment 1, there were priming effects in response tendencies for passives and in response latencies for actives. However, when participants' pre-existing preference for actives was altered in Experiment 2, syntactic priming occurred for both actives and passives in response tendencies as well as in response latencies. This is the first investigation of the effects of structure frequency on both response tendencies and latencies in syntactic priming. We discuss the implications of these data for current theories of syntactic processing.

## Introduction

We repeat all kinds of linguistic units when we speak: words, phrases and even syntactic structures [1]. The tendency to use similar syntactic structures across sentences is called structural or syntactic priming [2]. When speakers produce a given structure in one sentence on a prime trial (e.g., a passive sentence: *‘The boy is kissed by the girl’*), the chance of producing the same structure on a subsequent, target trial increases (e.g., ‘*The woman is hugged by the man*’).

Syntactic priming provides a window into syntactic processing and therefore it allows testing different theories. There are two influential theories of syntactic processing in language production. The implicit learning theory [3], [4] proposes that syntactic persistence occurs through implicit error-based learning. This theory argues for a system in which sentence structures are assembled through the construction of abstract syntactic frames into which lemmas are then inserted. Since implicit learning takes place outside the mental lexicon, this theory does not predict syntactic priming effects to be boosted by lexical repetition. An alternative theory is the residual activation theory [5], [6] which explains syntactic persistence in terms of a short-term memory or activation effect of syntactic frames which are tied to the lexicon and determine word order. This entails that syntactic processing is lexically driven and that syntactic priming effects will be boosted when the head of the construction (e.g., the verb for transitive sentences) is repeated.

Numerous language production studies have investigated syntactic priming effects for transitive sentences by measuring response tendencies, i.e. the frequency of speakers choosing one structure over an alternative structure on target trials. These studies found evidence for syntactic priming of transitives in both English [2], [7], [8], [9], [10], [11] and Dutch [12], [13]. However, while these priming effects have been shown repeatedly for passive sentences, comparable effects for active sentences are either absent [2], [9], [13] or smaller than for passives [2], [12]. A ceiling effect in the baseline frequency of producing actives may explain the absence or weakness of syntactic priming for actives in response tendencies: in Dutch written discourse, the proportion of active transitives is about 92% and, in English, about 88% [14].

Syntactic priming effects for active transitives may, however, be revealed in response latencies, which may not suffer from such a ceiling effect. Levelt and Kelter [15] suggested that the function of syntactic persistence may be to promote fluency and speed of sentence production and to reduce processing costs for the speaker, but very few studies have investigated priming effects in response latencies (for datives [16]; for noun phrases [17], [18]).

The implicit learning theory of syntactic priming [3], [4] is a theory about structure selection and does not make specific predictions about response latency effects. The residual activation theory as put forward by Pickering and Branigan [6] does also not make specific predictions about response latency effects. However, others have derived the prediction from this model that response latency effects should mirror response tendency effects [16]. This assumes that the activation in syntactic units determines not only choice but also selection speed. In the case of transitives, activation in a syntactic unit influences word order by activating the agent or patient as subject of the sentence. Residual activation makes it more likely for the same units to reach the selection threshold and be used again, changing response tendencies on target trials. Under the assumption that response tendencies and response latencies are both outcomes of the same mechanism, thresholds are reached faster when specific structures are repeated, resulting in faster response latencies.

In the present study we investigated syntactic priming of transitives in Dutch spoken language production using a picture description paradigm. We simultaneously measured response tendencies and response latencies. In Experiment 1 we explored the hypothesis that actives can be syntactically primed and that syntactic repetition of actives would result in faster response latencies. We hypothesized that in response tendencies there would an apparent syntactic priming effects for passives while the effect for actives may be obfuscated due to a ceiling effect in the baseline frequency of actives. We expected to see syntactic priming effects for actives as well as passives in speech onset latencies. If, however, the lack of response tendency effects for actives is not due to a ceiling effect but due to actives being less prone to syntactic priming, effects for actives should also be absent in the response latencies.

## Experiment 1

### Materials and Methods

#### Participants

Thirty native Dutch speakers (15 male/15 female, mean age of 23 years with SD 3.9) gave written informed consent prior to the experiment (as approved by the local ethics committee Commissie Mensengebonden Onderzoek Region Arnhem-Nijmegen) and were compensated for their participation.

#### Materials

Our stimulus pictures depicted 36 transitive events such as *kissing*, *helping*, or *strangling* with the agent and patient of this action (Appendix S1). The pictures elicited transitive sentences. Each event was depicted with two pairs of adults and one pair of children. There was one male and one female actor in each picture, and each event was depicted with each of the two actors serving as the agent. The position of the agent (left or right) was randomized.

Each transitive picture had three versions: one grayscale version and two color-coded versions with a green and a red actor (which elicited either an active or passive transitive - see task description). Fillers elicited either intransitive sentences, depicting events such as *running*, *singing*, *bowing* with one actor (in grayscale, green or red) or locative sentences, showing events such as *standing*, *sitting*, *lying* with either two objects or one actor and one object (either grayscale or color-coded to elicit a locative state or a frontal locative).

We pretested the materials to verify whether the depicted actions were clear and to measure which verb was most commonly used to describe each action. In the experiment this verb was presented preceding the picture.

#### Task and design

**Figure 1:**
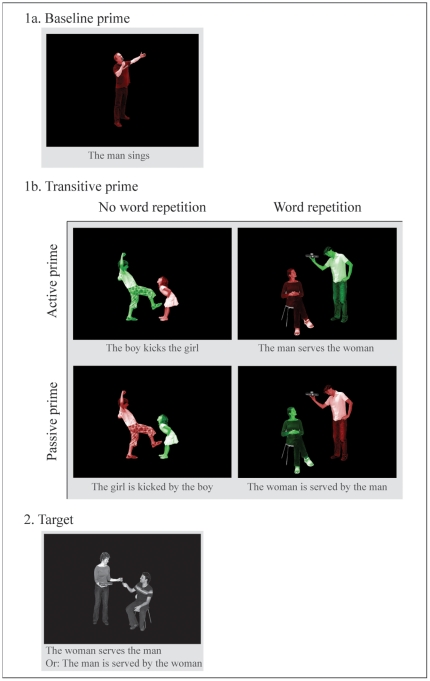
Design Experiment 1. Each trial consisted of a color-coded prime (1a. or 1b.) and a grayscale target (2.). On baseline trials (1a. followed by 2.) primes were intransitive or locative sentences, so that we could measure the baseline frequency of using active and passive transitives. On transitive priming trials (1b. followed by 2.) we measured the syntactic priming effect for transitive sentences in four conditions. Transitive primes could be active (top row) or passive (bottom row). Furthermore, there could be no word repetition (left column) or word repetition (right column) between prime and target. The sentences participants produced responding to the pictures are inserted for clarity. (Consent for publication was obtained from the actors depicted in these stimuli.)

The task and design of this experiment were adapted from Menenti et al. [19] and are illustrated in Figure 1. Participants were instructed to describe pictures with one sentence, naming the green actor before the red actor if the actors were depicted in color. If the actors were not depicted in color then participants did not have to pay attention to the order of mentioning the two actors and could therefore produce either an active or a passive sentence.

Each trial consisted of a prime followed by a target. Primes were pictures in which actors were color-coded for the order of precedence in the sentence, allowing us to manipulate the syntactic structure participants would produce (example 1a and 1b in Figure 1). A grayscale target eliciting a transitive sentence immediately followed the prime (example 2 in Figure 1).

There were two types of trials: baseline trials and transitive priming trials. On baseline trials, primes were intransitive or locative sentences (1a in Figure 1) so that we could measure the baseline frequency of producing active and passive transitives on subsequent targets. On transitive priming trials we measured the syntactic priming effect in four conditions (1b in Figure 1), resulting from a manipulation of prime structure (active versus passive), fully crossed with a manipulation of word repetition (no word repetition versus word repetition between prime and target). With the latter manipulation we investigated the influence of repeating words on syntactic priming effects. Note that in the word repetition conditions not only the verb, but also the actors are repeated. Preserving word order in these conditions implies reversing the thematic roles in the sentence. Syntactic priming effects are then unaffected by thematic role priming.

As in Menenti et al. [19], there were also successive transitive sentences for which words as well as sentence-level meaning were identically repeated. Since these trials are not relevant for the issues at stake here, they are not included in the analysis (including these trials in the analysis does not change the effects or their significance levels).

Intransitive *(‘The man sings’)* and locative *(‘The bottle stands on the table’)* sentences served as fillers, such that over the whole experimental list half of the items elicited transitives and half of the items did not.

In total, each experimental list contained 72 baseline trials and 24 trials in each of the 4 transitive priming conditions. We generated counterbalanced lists so that each target picture occurred once with a baseline prime, once with an active prime and once with a passive prime across each triplet of experimental lists.

#### Procedure

**Figure 2:**
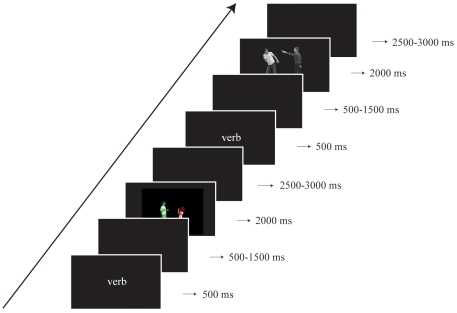
Procedure Experiment 1 and 2. Each trial consisted of the following events: a verb was presented in its infinitive form and after a jittered interval the prime picture was presented. After a jittered interval a verb was again presented, followed by the next jittered interval and a target picture. After another jittered interval the next trial started. (Consent for publication was obtained from the actors depicted in these stimuli.)

Participants received ten practice trials at the beginning of the experimental session. The actual experiment lasted 50 minutes. Figure 2 illustrates the sequence of events on each trial. Participants' responses were recorded and a voice key measured response latencies from picture presentation.

Responses were manually coded as active or passive. Target responses were considered for analysis only if 1) the correct structure was used on the prime trial and 2) both actors were named accurately and the verb was used correctly on both prime and target trial. Debriefing showed that participants were unaware of the purpose of the experiment.

### Results

#### Response tendencies

We excluded 6.5% (330 out of 5040) of the target responses because they were incorrect (criteria are described under ‘Procedure’). We analyzed the responses using mixed-effects logit models [20], [21] in R [22]. Coefficient estimates are included in the text only when a full summary is not included in the tables. Target responses were coded as 0 for actives and 1 for passives.

**Figure 3:**
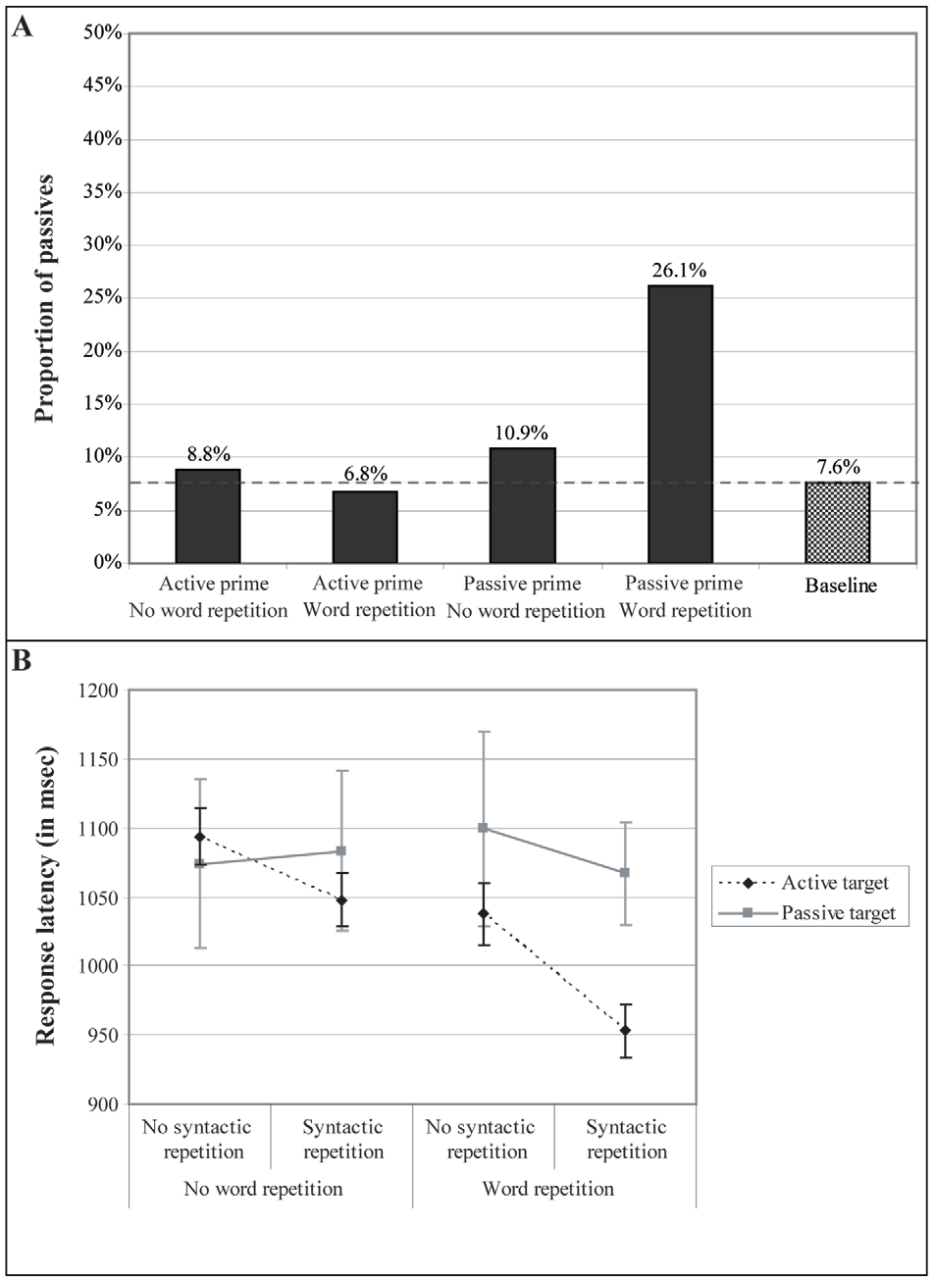
Results Experiment 1. A) Response tendency results: the proportion of passive transitives is illustrated for each condition, and B) Response latencies: mean response latencies and standard errors for each condition.

Figure 3a summarizes the proportion of passive responses. When we exclude the data from the baseline condition, we can fit a model with the predictors ‘Prime structure’ and ‘Word repetition’. We modeled random subject and item effects by including a random intercept and random slopes of ‘Prime structure’ and ‘Word repetition’ for subjects and a random intercept for items (this is the maximal random effect structure justified by model comparison). This shows that prime structure (p>.52) did not and word repetition (p<.015) did predict the response tendencies. Also the interaction between prime structure and word repetition predicted the response tendencies (p<.001) (upper part of table 1). To investigate then whether prime structure and word repetition change the response tendencies compared to the baseline proportion of passives versus actives, a predictor with ‘Condition’ with five levels was added such that the baseline condition was included in the intercept and contrasted with the four conditions which result from fully crossing ‘Prime structure’ and ‘Word repetition’ (see bottom of Table 1). Random subject and item effects were modeled by including a random intercept and slope of ‘Condition’ for subjects and a random intercept for items (this is the maximal random effect structure justified by model comparison). The negative estimate for the intercept indicates that in the baseline condition actives were more frequent than passives. Active primes affected the response tendencies when words were repeated (p<.04) (the negative coefficient indicates that more actives were produced relative to baseline) but not when words were not repeated (p>.09). The response tendencies after an active prime without word repetition differed significantly from the response tendencies after an active prime with word repetition (β = −0.86, p<.006). Passive primes affected response tendencies compared to baseline both when we compared the baseline to passive primes with word repetition (p<.006) and when we compared the baseline to passives primes without word repetition (p<.001) (the positive coefficient indicates that more passives are produced relative to baseline). The response tendencies after a passive prime with word repetition differed significantly from the response tendencies after a passive prime without word repetition (β = −1.08, p<.001).

**Table 1:** Summary of fixed effects in the mixed logit model for the response tendencies in Experiment 1.

Predictor	coefficient	*SE*	*Wald Z*	*p*
Excluding the baseline condition (N = 2480, log-likelihood = −769)				
Intercept	−3.04	(0.29)	−10.47	<.001 ***
Prime	0.17	(0.27)	0.64	>.52
Word repetition	−0.68	(0.28)	−2.43	<.015 *
Prime by Word repetition	1.91	(0.30)	6.38	<.001 ***
Including the baseline condition in the intercept (N = 4710, log-likelihood = −1261)				
Intercept (Baseline)	−3.36	(0.29)	−11.66	<.001 ***
Active prime - No word repetition	0.32	(0.19)	1.73	>.09
Active prime – Word repetition	−0.54	(0.26)	−2.08	<.04 *
Passive prime - No word repetition	0.47	(0.17)	2.74	<.006 **
Passive prime – Word repetition	1.55	(0.26)	5.97	<.001 ***

#### Response latencies

We excluded 7.5% of correct responses on transitive priming trials (195 out of 2580) because they contained other sounds which triggered the voice key before speech onset or because they were two standard deviations below or above the mean calculated per subject and per condition [23]. We created a post-hoc independent variable ‘Syntactic repetition’ based on the relationship between prime structure and the structure of the participant's target response. Response latencies were analyzed using mixed-effects linear models [21], [24] in R. (Results are identical when response latencies are analyzed with a repeated-measures ANOVA. Although mixed-effects linear models are less often applied, they are better suited for use with post-hoc independent variables).

Figure 3b summarizes the response latency data. The fixed effects of the best model fit for these data are summarized in Table 2. As reference conditions we used: active targets, no syntactic repetition and no word repetition. We included the random intercept and slope of ‘Syntactic repetition’ and ‘Word repetition’ for subjects, and the random intercept for items (this is the maximal random effect structure justified by model comparison). Syntactic repetition significantly speeded up response latencies (p<.001), as did word repetition (p<.001). However, the interaction between syntactic repetition and target structure indicates that the effect of syntactic repetition was different for passives than for actives (p<.02). To further investigate this interaction, we constructed the factor ‘Condition’ with four levels: actives with syntactic repetition, actives without syntactic repetition, passives with syntactic repetition, and passives without syntactic repetition (we estimated this model including the random intercept and slope of ‘Word repetition’ for subjects, and the random intercept for items). When active targets without syntactic repetition were included in the intercept and hence contrasted to the other levels of the ‘Condition’ factor, the analysis showed that the response latencies for active targets were significantly faster with syntactic repetition than without syntactic repetition (β = −56.63, p<.001). When passive targets without syntactic repetition were included in the intercept and contrasted to the other levels of this factor, the analysis showed that for passive targets syntactic repetition did not significantly predict response latencies (β = 31.53, p>.4).

**Table 2:** Summary of fixed effects in the mixed linear model for the response latencies in Experiment 1.

Predictor	coefficient	*SE*	*t value*	*df*	*Pr(>|t|)*
Intercept	1046.06	57.65	18.14	2020	<.001 ***
Target structure	−0.62	33.09	−0.02	331	>.98
Syntactic repetition	−56.02	17.29	−3.24	331	<.001 ***
Target structure by Syntactic repetition	89.94	39.46	2.28	331	<.02 *
Word repetition	−69.90	16.54	−4.23	331	<.001 ***

Note: N = 2385, log-likelihood = −16970. Because Markov chain Monte Carlo sampling [24] is not yet implemented for models with random slopes we cannot provide *p*-values based on the posterior distribution. The *p*-values based on the *t*-distribution should therefore only be interpreted with caution. (They were calculated using the package nlme [22]).

Although word repetition significantly speeded up the response latencies, it is noteworthy that there was no three-way interaction between word repetition, syntactic repetition, and target structure: including this interaction did not improve the fit of the model (χ^2^_3_ = 4.55, p>.21).

### Discussion

In Experiment 1 we investigated syntactic priming of transitive sentences in Dutch spoken language production using a picture description paradigm. When syntactic priming is not helped by additional word repetition, we found syntactic priming effects for passives but not actives in the response tendencies and for actives but not passives in the response latencies. In the response tendencies however, word repetition did boost the syntactic priming effect and then not only the effect for passives but also the effect for actives reached significance. Word repetition did not affect priming in response latencies.

These results lend support to the idea that the initial preference ratio of two syntactic alternatives is an important determinant of syntactic priming. Transitive events can be described with active or passive sentences, though crucially, speakers have a strong preference for using actives instead of passives (in Experiment 1 the baseline frequency of actives was 92%). Due to this pre-existing bias, the tendency to select actives is at ceiling, so there is little room for active primes to increase this tendency. An effect of active primes on the response tendencies was however observed when syntactic priming was boosted by word repetition. That actives benefit from syntactic repetition was even more apparent in the response latencies: syntactically repeated actives are produced faster, irrespective of word repetition. This effect of syntactic priming on response latencies for actives had so far not been investigated.

For passives we found syntactic priming effects in response tendencies, replicating previous findings [12], [13]. Just like it is the case for actives, for passives the effect of syntactic priming on response latencies had so far not been investigated. We found that there was no latency benefit for repeated passives. Response tendencies and response latencies thus seem to have different sensitivities to the frequency of syntactic constructions. To investigate the role of the relative frequency of syntactic alternatives in determining syntactic priming effects further, we performed a second experiment.

There are in fact other differences between actives and passives than their relative frequency of occurrence. Passives are for instance stylistically marked, or used when there are pragmatic reasons to put the patient of the action in focus. To test whether the results of Experiment 1 are due to the difference between actives and passives in frequency of occurrence per se, or to another difference between actives and passives, we performed Experiment 2. In Experiment 2, we manipulated the relative frequency of occurrence of actives and passives by subjecting participants to a training session before the actual experiment started. During this training session we exposed participants to a pattern of experience with active and passive sentences. In one group the training maintained the pre-existing ratio for actives versus passives, while in another group this was reversed, so that the bias to produce actives instead of passives would become less strong. Kaschak [25] has demonstrated that such a manipulation affects the base rates of producing the two alternative constructions. If the difference in the effect of syntactic priming on response tendencies versus response latencies for actives and passives in Experiment 1 is indeed due to the difference in their relative frequency, a training session altering the relative frequency should affect the syntactic priming effects. When selection of passives is boosted we expect observable syntactic priming effects for actives as well as passives, both in the response tendencies and in the response latencies.

Another interesting outcome of Experiment 1 was that response tendencies and latencies did not only show differential effects for actives and passives, but also differed in the effect of word repetition on the magnitude of syntactic priming. Word repetition boosted priming effects in response tendencies but not in response latencies. It is important to note that because we aimed to investigate syntactic priming unaffected by thematic role priming in Experiment 1, all words (not just the verb) were repeated. The residual activation theory [5], [6] predicts that syntactic priming effects will be boosted when the head of the construction - in the case of transitives this is the verb - is repeated. Therefore, in Experiment 2 we manipulated repetition of the verb when other words in the sentence were not repeated. This allows us to compare our results to those of studies reported in the literature, which traditionally include a manipulation of verb repetition, but not repetition of verb and nouns at the same time.

## Experiment 2

In Experiment 2, we tested whether the different syntactic priming effects for actives and passives in response tendencies versus response latencies is indeed due to their relative frequency of occurrence. We submitted one group of participants, the experimental group, to a training session in which they had to produce 90% passive sentences and 10% active sentences. Participants then completed a task similar to that reported in Experiment 1. We expected that the training session alters participants' preference bias such that the selection of passives is boosted. Therefore, in this group we expected to find syntactic priming effects for actives as well as passives, both in response tendencies and response latencies. We submitted another group of participants, a control group, to a training session in which they had to produce 10% passives and 90% actives, maintaining the strong preference bias for actives. We hypothesized that in this group we would replicate the results of Experiment 1: we expected to find a syntactic priming effect for passives in the response tendencies and a priming effect for actives in the response latencies.

### Materials and Methods

#### Participants

Sixty native Dutch speakers (mean age 22 years with SD 3.07; with 30 males divided evenly over control and experimental group) gave written informed consent prior to the experiment (as approved by the local ethics committee Commissie Mensengebonden Onderzoek Region Arnhem-Nijmegen) and were compensated for their participation.

#### Materials and task

Materials were largely identical to those used in Experiment 1. Additional transitive pictures were created so there were pictures of 41 transitive events in total (Appendix S1). Like in Experiment 1, fillers elicited either intransitive sentences or locative sentences.

The picture description task was identical to the task in Experiment 1: participants were instructed to describe pictures with one sentence, naming the green actor before the red actor if these were depicted in color. If the actors were not depicted in color then participants did not have to pay attention to the order of mentioning the characters in the sentence.

#### Design

Preceding the experiment, participants completed a training session, supposedly to practice the task, during which they produced descriptions of transitive color-coded pictures. The proportion of actives versus passives which was produced during this training session was manipulated between participants. The control group produced active descriptions in 90% of all trials and passive descriptions in 10% of all trials. The experimental group produced active descriptions in 10% of all trials and passive descriptions in 90% of all trials. In this session, pictures depicted one of 10 transitive verbs *(pelt, kiss, make up, punish, transport, scare, embrace, drag, draw, strangle)*. For each of these 10 verbs there were 10 pictures. The verbs were different from the 31 transitive verbs encountered later during the syntactic priming experiment.

**Figure 4:**
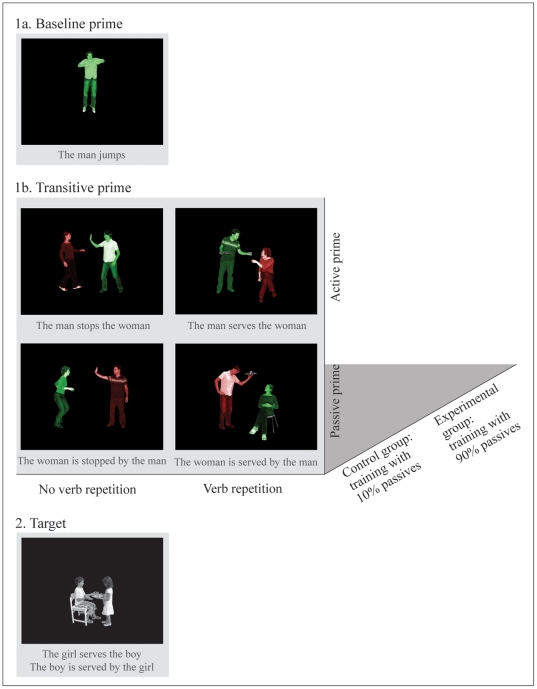
Design Experiment 2. Preceding the experiment, participants completed a training session. The type of training session was manipulated between participants: one group received a training block with 10% passives (the control group) and a second group received a training block with 90% passives (the experimental group). During the actual experiment, each trial consisted of a color-coded prime (1a. or 1b.) and a grayscale target (2.). On baseline trials (1a. followed by 2.) primes were intransitive or locative sentences, so that we could measure the baseline frequency of using active and passive transitives. On transitive priming trials (1b. followed by 2.) we measured the syntactic priming effect for transitive sentences in four conditions. Transitive primes could be active (top row) or passive (bottom row). Furthermore, there could be no verb repetition (left column) or verb repetition (right column) between prime and target. The sentences participants produced responding to the pictures are inserted for clarity. (Consent for publication was obtained from the actors depicted in these stimuli.)

In the experiment, like in Experiment 1, each trial consisted of a color-coded prime followed by a grayscale target, and there were two types of trials: baseline trials and transitive priming trials (Figure 4). During transitive priming trials we measured the syntactic priming effect in four conditions, resulting from a manipulation of prime structure (active vs. passive), fully crossed with a manipulation of verb repetition (no verb repetition vs. verb repetition between prime and target). With the latter manipulation we investigated the influence of repeating verbs on syntactic priming effects.

Each experimental list contained 48 baseline trials and 24 trials in each of the 4 transitive priming conditions. We generated counterbalanced lists so that each target picture occurred once with a baseline prime, once with an active prime and once with a passive prime across three different experimental lists. Over the whole experiment, half of the items elicited transitives and half of the items elicited other structures.

Participants first saw 100 pictures during the training session and then 480 pictures during the actual experiment. Each experimental list was presented to a participant who had a training session with 10% passives and to a participant who had a training session with 90% passives.

#### Procedure

The training session was portrayed to the participants as a practice session preceding the actual experiment. We told them this practice session would give them a chance to familiarize themselves with the task. The training session lasted 10 minutes. The actual experiment lasted 48 minutes and the procedure followed the one described for Experiment 1 (see also Figure 2).

### Results

#### Response tendencies

We excluded 7.7% of the target responses (669 out of 8640; in group 1: 321 out of 4320 (7.4%) and in group 2: 348 out of out of 4320 (8.1%)) because they were incorrect. We analyzed the responses using mixed-effects logit models in R [20], [21]. Active targets were coded as 0 and passive targets as 1.

**Figure 5:**
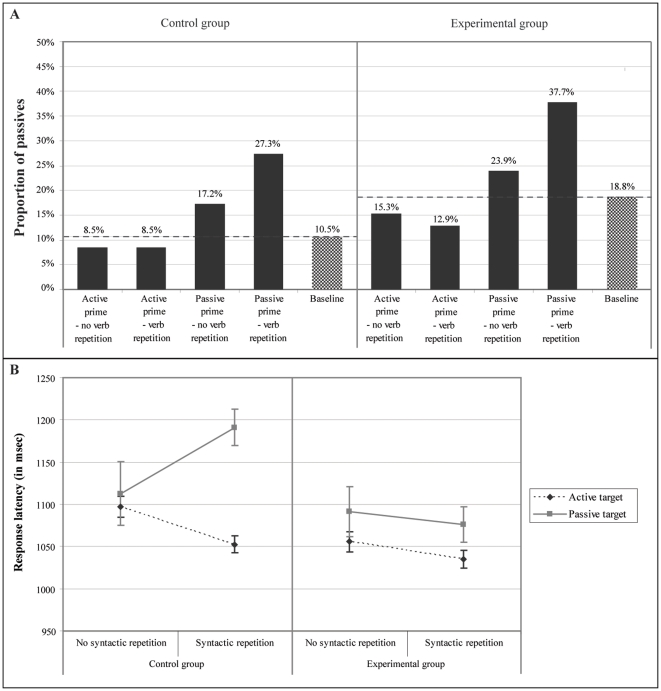
Results Experiment 2 for the control group (left panel) and experimental group (right panel). A) Response tendency results: the proportion of passive transitives is illustrated for each condition, and B) Response latencies: mean response latencies and standard errors for each condition.

Figure 5a summarizes the proportion of passive responses. The between-group manipulation of structure frequency in the training session produced the effect we expected: in the experimental group the production of passives was boosted compared to the control group. The preference bias changed from 10.5% passives in the baseline condition in the control group to 18.8% passives in the baseline condition in the experimental group.

When we exclude the data from the baseline condition, we can fit a model with the predictors ‘Prime structure’, ‘Verb repetition’ and ‘Group’ (upper part of Table 3). Random subject and item effects were modeled by including a random intercept and slope of ‘Prime structure’ for subjects and a random intercept for items (this is the maximal random effect structure justified by model comparison). The negative intercept indicates that actives were overall more preferred than passives. Group (p<.008), Prime structure (p<.001) and the interaction between Prime structure and Verb repetition (p<.001) were significant predictors of response tendencies.

**Table 3:** Summary of fixed effects in the mixed logit model for the response tendencies in Experiment 2.

Predictor	coefficient	*SE*	*Wald Z*	*p*
For the control and experimental group taken together, excluding the baseline condition (N = 5254, log-likelihood = −2141)				
Intercept	−2.87	0.22	−13.24	<.001 ***
Prime	0.82	0.17	4.88	<.001 ***
Verb repetition	−0.16	0.14	−1.18	>.24
Group	0.61	0.23	2.66	<.008 **
Prime by Verb repetition	0.93	0.17	5.53	<.001 ***
For the control group, including the baseline condition in the intercept (N = 3972, log-likelihood = −1334)				
Intercept (Baseline)	−2.78	0.23	−12.06	<.001 ***
Active prime – No verb repetition	−0.42	0.21	−1.97	<.049 *
Active prime - Verb repetition	−0.27	0.20	−1.37	>.16
Passive prime - No verb repetition	0.71	0.16	4.56	<.001 ***
Passive prime - Verb repetition	1.44	0.20	7.13	<.001 ***
For the experimental group, including the baseline condition in the intercept (N = 3999, log-likelihood = −1822)				
Intercept (Baseline)	−1.79	0.19	−9.60	<.001 ***
Active prime - No verb repetition	−0.42	0.16	−2.63	<.009 **
Active prime - Verb repetition	−1.02	0.21	−4.81	<.001 ***
Passive prime - No verb repetition	0.36	0.14	2.49	<.01 *
Passive prime - Verb repetition	1.07	0.23	4.59	<.001 ***

To investigate whether prime structure and word repetition change the response tendencies compared to the baseline proportion of passives versus actives in each group, we then analyzed the data of the control group and the experimental group separately, and, analogous to the analyses of Experiment 1, we included the baseline condition in the intercept (middle and bottom part of Table 3). In the control group we modeled random subject and item effects by including a random intercept and random slope of ‘Condition’ for subjects and a random intercept for items (this is the maximal random effect structure justified by model comparison); in the experimental group we modeled random subject and item effects by including a random intercept and random slope of ‘Condition’ for subjects as well as for items (this is the maximal random effect structure justified by model comparison).

The negative estimate for the intercept in the control group and in the experimental group indicates that actives were more frequent than passives in both groups in the baseline condition. While in the control group actives were produced arguably more often following an active prime compared to baseline (no verb repetition: p<.050, verb repetition: p>.16), in the experimental group actives were produced significantly more often following an active prime compared to baseline (no verb repetition: p<.009, verb repetition: p<.001). Following a passive prime, on the other hand, more passive targets were produced compared to baseline both in the control group (no verb repetition: <.001, verb repetition: <.001) and in the experimental group (no verb repetition: <.01, verb repetition: <.001).

#### Response latencies pre-experimental training session

In the control group 47 out of 3000 (1.6%) responses during the training session were incorrect. In the experimental group 36 out of 3000 (1.2%) responses were incorrect. Paired samples t-tests on the response latencies of the correct responses revealed that in the control group actives were produced 223.5 ms faster than passives (t_29_ = −9.642, p<.001) and in the experimental group passives were produced 94.6 ms faster than actives (t_29_ = 3.240, p<.003).

#### Response latencies experimental session

We excluded 5.4% of correct responses on transitive priming trials (284 out of 5254; in the control group: 152 out of 2614 (5.8%) and in the experimental group: 132 out of out of 2640 (5.0%)) because they were coded as containing other sounds which triggered the voice key before speech onset or because they were two standard deviations below or above the mean calculated per subject and per condition. Based on participants' target responses we created a post-hoc independent variable ‘Syntactic repetition’. Response latencies were analyzed using mixed-effects linear models in R [21], [24].

Figure 5b summarizes the response latency data. We first analyzed the data of the control and experimental group together to investigate the effect of the between-group manipulation of the training session. The fixed effects of the best model fit are summarized in the upper part of Table 4. We modeled between group random subject and item effects by including a random intercept (this is the maximal random effect structure justified by model comparison). In this model estimation, passive targets, no syntactic repetition, no verb repetition and the experimental group are taken as reference, and, importantly, passive targets are included in the intercept. We took passive targets as the reference because we primarily set out to investigate the effect of pre-experimental training on the latencies for passives. For passive targets syntactic repetition slowed down response latencies (p<.047), however, and crucially, the interaction between syntactic repetition and group indicates that for passive targets the effect of syntactic repetition is different in the two groups (p<.012). While in the control group syntactic repetition increased the latencies for passives, in the experimental group syntactic repetition decreased the latencies for passives. Additionally, the effect of syntactic repetition was different for active and passive targets (p<.001) and there was also a three-way interaction between syntactic repetition, target structure, and group (p<.004).

**Table 4:** Summary of fixed effects in the mixed linear model for the response latencies in Experiment 2.

Predictor	coefficient	MCMC mean	HPD95 lower	HPD95 upper	*pMCMC*	*Pr(>|t|)*
For the control and experimental group taken together (N = 4970, log-likelihood = −34871)						
Intercept (passives)	1079.53	1071.21	−242.22	2295.83	<.08	<.001 ***
Target structure	−5.26	−5.86	−62.22	49.44	>.84	>.85
Syntactic repetition	62.17	61.97	−0.78	120.70	<.047	<.047 *
Group	−24.88	−8.42	−1769.46	1907.81	>.93	>.65
Target structure by Syntactic repetition	−117.15	−116.94	−179.74	−49.99	<.001	<.001 ***
Target structure by Group	−20.55	−20.09	−92.12	50.13	>.58	>.57
Syntactic repetition by Group	−100.23	−99.86	−174.14	−21.02	<.010	<.012 *
Target structure by Syntactic repetition by Group	124.01	123.74	44.24	210.29	<.003	<.004 **
For the control group (N = 2462, log-likelihood = −17148)						
Intercept	1074.57	1073.83	1013.07	1131.79	<.001	<.001 ***
Target structure	1.23	2.21	−49.93	56.44	>.94	>.96
Syntactic repetition	−55.30	−55.25	−76.64	−33.49	<.001	<.001 ***
Target structure by Syntactic repetition	119.49	19.23	59.14	180.38	<.001	<.001 ***
For the experimental group (N = 2508, log-likelihood = −17723)						
Intercept	1027.27	1026.88	980.40	1075.76	<.001	<.001 ***
Target structure	27.63	27.76	−1.04	55.10	<.051	<.050 *
Syntactic repetition	−33.03	−32.79	−55.22	−10.21	<.005	<.005 ***

Note: Listed are the model estimates and the mean estimate across Markov chain Monte Carlo samples for the coefficients, with the upper and lower 95% highest posterior density intervals and *p*-values based on the posterior distribution and the *t*-distribution (with upper bound degrees of freedom) [24].

Therefore, we investigated the group effect on the latencies for actives next. In order to do this, we estimated the same model but this time we chose active targets as the reference and included active targets in the intercept. The analysis then revealed a main effect of syntactic repetition for actives (β = −54.98, p<.001) but no interaction between syntactic repetition and group for this structure (β = 23.78, p>.16). This means that for active targets syntactic repetition increased the response latencies. In addition, unlike for passive targets, the syntactic repetition effect for active targets was not modulated by the training session (i.e., there was no reliable difference between the control and experimental groups).

Including a predictor for verb repetition (as a main effect: χ^2^_1_ = 0.23, p>.63; or interacting with the other predictors: χ^2^_8_ = 8.17, p>.42) did not improve the model fit of the response latency data of Experiment 2.

To further examine the effect of the between-group manipulation, we analyzed the data of the control group and the experimental group separately. In both groups we modeled random subject and item effects by including a random intercept (this is the maximal random effect structure justified by model comparison). The analysis of the control group (middle part of Table 4) revealed that syntactic repetition decreased response latencies (p<.001), but this effect depended on whether the target structure was active or passive (p<.001). Therefore, in a similar manner to Experiment 1, a factor with four levels was constructed, making it possible to contrast syntactic repetition to no syntactic repetition for active and passive targets separately. For active targets, response latencies were shorter for syntactic repetition compared to no syntactic repetition (β = −55.30, p<.001), while for passive targets, response latencies were longer for syntactic repetition compared to no syntactic repetition (β = 64.19, p<.03). The analysis of the experimental group (bottom part of Table 4) on the other hand, revealed that syntactic repetition decreased response latencies for both target structures taken together (p<.005). Interestingly, in the experimental group, allowing an interaction of syntactic repetition with target structure did not improve model fit (χ^2^_1_ = 0.13, p>.72).

### Discussion

In Experiment 2 we aimed to further investigate the role of speakers' pre-existing bias in determining syntactic priming effects of actives versus passives. In the control group of participants, who had a training session maintaining the strong pre-existing bias towards actives, we replicated the syntactic priming effects of Experiment 1. In this group there was a syntactic priming effect for passives in the response tendencies and for actives in the response latencies. In the experimental group however, who had a training session altering the preference bias such that the base rate selection of passives was boosted, we found syntactic priming effects for both structures in the response tendencies as well as the response latencies.

Experiment 2 thus confirms that the preference ratio of two syntactic alternatives is a crucial determinant of syntactic priming, and moreover shows that this bias is dynamic and subject to learning. A relatively short training block which gave participants experience with a high proportion of passive sentences substantially changed their preference bias. The experience during this training block (90% passives and 10% actives) was opposite to their lifelong experience (10% passives and 90% actives). This recent experience added to, but evidently did not replace, their lifelong experience.

With respect to actives, the results of Experiment 2 seem to confirm that a ceiling effect in the baseline frequency may obfuscate response tendency effects for this syntactic alternative. The training session had a reliable impact on the response tendency results for actives. In the control group (where the baseline preference for actives was ∼90%), active primes again seemed to slightly affect the response tendencies; the effect just reached significance when there was no verb repetition and did not reach significance when there was verb repetition. However, in the experimental group (where the baseline preference for actives was ∼80%) the response tendency effects for actives were much stronger than in the control group, although they were still smaller than for passives. Additionally, Experiment 2 confirmed that there is a reliable and consistent response latency benefit of syntactically repeating the more preferred alternative (i.e., the active).

For passives, the training session had a reliable impact on the response latency effects. In the control group, there was no facilitation of the response latencies when passive structures were repeated - in fact, the results showed increased response latencies. This differs from the finding in Experiment 1 where there was no observable latency effect for syntactically repeated passives. Future experiments need to investigate possible reasons for the difference in results. One possible reason may be the training block that the control group of Experiment 2 had to complete. The ratio between actives and passives in this training block was similar to the one in daily life. But unlike in daily life, these transitive sentences were not mixed with other syntactic structures, thus putting the frequency difference between actives and passives in the spotlight and enhancing the effect. In the experimental group of Experiment 2 there was a facilitation effect in the response latencies for active and passive structures taken together. There was no evidence of an interaction between the effect of syntactic repetition and whether the syntactic structure was active or passive. In this group, the relative frequency of passives was boosted: actives were preferred over passives (∼20% passives were produced in the baseline condition) but less so than in the control group (where ∼10% passives were produced in the baseline condition).

As a final point, in the present experiment we included a manipulation of verb repetition while the other words in the sentence were not repeated. Although in Experiment 1 we included full word repetition, the results of this manipulation in the two experiments are comparable: verb repetition and, more generally, repetition of content words boosts syntactic priming effects in response tendencies, but not in response latencies. Repetition of the nouns together with repetition of the verb, however, leads to a lexical priming effect in response latencies, but repetition of the verb alone does not.

## Discussion

In the present set of experiments we investigated syntactic priming of transitive syntactic structures in Dutch spoken language production using a picture description paradigm. We simultaneously measured response tendencies and response latencies. In Experiment 1, we found that syntactic priming readily affects the response tendencies for passives, while in the response latencies there is only facilitation for syntactically repeated actives. That the difference between actives and passives in these syntactic priming outcomes is related to speaker's preference bias for actives was confirmed by Experiment 2. Following a training session maintaining participants' strong preference bias for actives, we replicated the findings of Experiment 1. However, following a training session altering participants' preference bias such that the base rate of passives is boosted, we found syntactic priming effects for both structures in the response tendencies as well as the response latencies.

For the analyses of the response latencies, we did not manipulate the factor ‘Syntactic repetition’ but constructed it on the basis of the participants' own responses. Therefore, we can strictly speaking only draw correlational and not causal conclusions regarding the relationship between ‘Syntactic repetition’ and the response latencies. However in two different studies in which we did manipulate ‘Syntactic repetition’ as a factor [19], [26], we also found response latency benefits for actives and not passives, indicating that ‘Syntactic repetition’ causes the response latency effects and not the reverse.

Very few studies have investigated response latency effects of syntactic priming [16], [17], [18] and these did not yet take preference biases into account. Smith and Wheeldon [17], [18] found latency effects for noun phrases, structures for which detailed information on preference biases is unknown. Corley and Scheepers [16] found syntactic priming evidence for English datives in response tendencies as well as response latencies (note however that they only found reliable effects in the verb repetition condition). For datives, preference biases are verb specific [27]. Corley and Scheepers [16] used a large set of materials [6] containing verbs with a prepositional object preference as well as verbs with a double dative object preference. Thus, they collapsed the effects of primes with prepositional object preference verbs and double dative object preference verbs. Teasing these apart may reveal the effects of verb-specific alternation biases on the strength of syntactic priming on response tendencies [28] and also latencies.

The preference ratio of two syntactic alternatives is a crucial determinant of syntactic priming effects. In response tendencies, not only for active and passive transitives but also for many other structural alternatives, priming with the less preferred structure shows stronger syntactic priming effects [28], [29], [30], [31]. This has been described in the inverse-preference account: learning, displayed as effects of priming on response tendencies, is a function of the degree of preference [32]. This is compatible with findings showing that the syntactic system is probabilistic in nature, since the effect of syntactic priming on response tendencies is sensitive to prime surprisal (surprisal is the inverse of probability) [33]. In other words, the strength of effects on response tendencies is inversely correlated with the degree of preference for the prime structure [32] or the extent to which the prime structure was expected [33]. Both proposals are related to the implicit learning theory [3], which specifies that the larger prediction error accompanying less preferred prime structures will lead to larger changes in internal representations and larger effects on response tendencies. In our experiments we found an inverse-preference effect in the response tendencies for transitives. While passive primes reliably and consistently affected the response tendencies, actives primes had a small or absent effect. In Experiment 2, when the preference ratio between actives and passives was less unbalanced and the frequency of passives boosted, there were larger syntactic priming effects in response tendencies for actives than in the control group of Experiment 2 and in Experiment 1.

While error-based implicit learning, inverse-frequency and surprisal accounts can explain the response tendency effects, in their current form these views are not able to explain the response latency results. We have shown in two experiments that there is a convincing facilitatory effect in the response latencies when the *more preferred* syntactic alternative, the active transitive, is repeated. For the less preferred syntactic alternative, the passive transitive, effects on response tendencies are not necessarily accompanied by a response latency benefit. Only when the bias against the less preferred alternative is sufficiently weak, a response latency effect prevails. An important conclusion we can therefore draw is that the response latency effects of syntactic priming do not mirror the response tendency effects.

In sum, we have observed that syntactic priming affects the less frequent, unpreferred construction (i.e. passive) and the more frequent, preferred construction (i.e. active) in different manners: it increases the frequency of the unpreferred alternative and decreases the response latency of the preferred alternative. This dichotomy presents a challenge to the field and to existing theories of syntactic priming: both the implicit learning theory [3] and the residual activation theory [5], [6] are currently underspecified with regards to response latency effects of syntactic repetition (see introduction). Here, we present a tentative model of our findings -a model partly based on spreading activation and inhibition (competition) between syntactic alternatives. In the next section we describe the model in more detail. We proceed from rather standard assumptions regarding the make-up and functioning of neurons in computational neural network models [34], [35]. The model could be computationally implemented in future work to test its performance.

### A competition model of syntactic priming

We assume that grammatical encoding of a transitive event proceeds in two sequential stages: (1) *a selection stage*, during which one of the alternative syntactic constructions is selected, and (2) *a planning stage*, during which production of the selected construction is prepared. We now describe in more detail the processes that take place in each stage.

#### Selection stage

Whether the conceptual representation of a perceived event that includes a transitive action is grammatically encoded in Active or Passive Voice, depends on, among other things, the current levels of activation of nodes (or neural assemblies) representing the Active Voice and the Passive Voice constructions. The activation level of the nodes can vary between 0 and 1. We assume that a node's “resting level” (or “base level”) of activation is positively correlated with its frequency of occurrence, in particular that the Active Voice node has a higher resting level than the Passive Voice node. Noise causes random fluctuation around the current average activation level even in the absence of other causal factors. (In an unprimed or resting situation, there are three influences enabling the Passive Voice to be selected occasionally as response choice despite its generally lower resting level activation: (1) random fluctuations due to noise, (2) feedforward activation from e.g. the semantic/conceptual representation of a picture during an experimental manipulation, and (3) feedback activation due to pragmatic factors (e.g., the patient of the transitive action being in the focus of attention).) Nodes transmit activation and inhibition ( = negative activation) to neighboring nodes in the network. There are inhibitory links between the two competing structural alternatives (with invariant stable weights, which we assume to be identical in either direction). The amount of inhibition transmitted to a competitor node is a positive function of the current level of activation. Activation coming in from neighboring nodes is added to the current activation of the node, and incoming inhibition is subtracted from the current activation level. Due to decay of activation, the current activation level decreases in each cycle by a small percentage.

The activation level of a node is updated during every processing cycle in the following way: the activation at the onset of cycle *t*+1 equals the activation at cycle *t* multiplied by the decay factor (e.g. .95), plus the activation coming in from neighboring nodes during cycle *t*, minus the inhibition from the competitor node during cycle *t*. A “squashing function” serves to keep the resulting activation between the upper and lower bounds of 1 and 0, respectively. Both nodes have two thresholds: a relatively low “excitation threshold” (e.g. at activation *a* = .3), and a relatively high “selection threshold” (e.g. *a* = .9). At activation levels below the excitation threshold, the nodes are “dormant”; that is, they do not emit any activation or inhibition. The resting levels of both competitor nodes are below the excitation threshold. For simplicity, we assume that the Active Voice and Passive Voice nodes have identical excitation thresholds, and identical selection thresholds. Reaching the selection threshold means that the node “fires” and that the corresponding construction (Active Voice or Passive Voice) is selected. After firing, the activation level drops gradually due to decay, finally returning to the dormant state and reaching the resting level of activation. The activation between the moments of firing and reaching the resting level is usually called “residual activation.”

The intention to describe a transitive event causes activation be to sent to both the Active and the Passive Voice nodes. This activation transmission continues until one of the competitor nodes reaches the selection threshold and fires. The time it takes to reach a selection threshold is determined by the time needed to solve the competition between the Active Voice node and the Passive Voice node. This time is negatively correlated with the difference in activation levels between the two competitors at the moment the competition starts: the higher the current activation of a node, the more inhibition it transmits to the competitor; and the lower the latter's activation, the less inhibition it can retort. Hence, the time needed to determine the winner of the competition *de*creases with an *in*creasing difference in activation levels between competitors, other things being equal. In other words, when priming increases the difference in activation levels between competitors (compared to the difference in base-level activation of the competitors), priming decreases the competition time. When priming decreases the difference in activation levels between competitors (compared to the difference in base-level activation of the competitors), it increases the competition time.

#### Planning stage

Once either the Active Voice or the Passive Voice is selected, production of the selected alternative is planned. We assume, in line with Levelt & Kelter [15], that priming reduces the planning time as an effect of practice.

#### Effects of syntactic priming

The model sketched above implies that the choice of a syntactic construction is determined exclusively during the selection stage. The response latency, on the other hand, depends on the course of events in both the selection stage and the planning stage: the durations of these stages contribute to the response latency as additive effects.

In reaction to an Active Voice prime (the more frequent construction), the following scenario unfolds. Since the relative frequency of active sentences is close to ceiling already prior to priming, the residual activation due to the priming manipulation cannot increase the selection frequency of the active construction to a large extent. Hence, the response tendency effect is very small or absent. The selection time may be slightly shorter (compared to the unprimed situation) since residual activation on the Active Voice node has increased the gap between the activation levels of the competitors. The planning stage, too, can proceed faster due to the practice effect. The effect on the selection time and the effect on the planning time are additive and result in faster response latencies.

Priming with a passive sentence (the infrequent alternative), temporarily increases the activation level of the Passive Voice node due to residual activation, thereby narrowing the gap with the competitor's activation level, or even reversing the momentary balance of power. As a consequence, the frequency of passives can increase. Crucially, the average time needed for the Passive Voice node to win the competition increases as well due to the reduced gap between activation levels of the competitors. The ensuing lengthening of the selection stage is not visible in the overall response latency because, during the planning stage, passives profit from the practice effect. The shortened planning time compensates fully (experiment 1) or partly (control group experiment 2) for the lengthened selection time.

#### Lexical influences on syntactic priming effects

On the assumption of a lexicalized grammar, e.g. [36], we hypothesize an activation-and-competition network with an Active Voice node and a Passive Voice node for every transitive verb. The Active Voice node of a particular verb inhibits the Passive Voice node of this particular verb but also activates the Active Voice nodes of other verbs (the same applies to Passive Voice nodes). The lexical boost in the response tendency results for passives could then be explained as follows: priming with a passive sentence temporarily increases the activation level of the Passive Voice node for the prime verb (as described) and also, but to a smaller extent, increases the activation level of the Passive Voice node for other verbs. For syntactic priming of actives, due to the ceiling effect in the base level activation, the selection stage can only be affected by syntactic priming and by word repetition to a small extent. The practice effect in the planning stage is unlikely to be influenced by verb repetition since for actives it is reasonable to assume that no more than only the first noun phrase is planned [37]. Possibly because any lexical boost in the selection stage is very small for actives and because a lexical boost is absent in the planning stage, the added effect of the two may not result in an apparent lexical boost of the response latency effects for actives.

### The implications for existing theories of syntactic priming

While our specific interpretation of these results in terms of a competition model is up for discussion, the results have important implications for existing theories on the mechanism behind syntactic priming. To be able to account for our findings, a theory of syntactic processing would have to comprise the following features: firstly, the syntactic priming mechanism would have to be sensitive to the preference bias of two syntactic alternatives. Secondly, the mechanism would have to be dynamic, such that the preference bias can change over time due to exposure to these syntactic alternatives. Thirdly, the mechanism would have to be able to explain that effects on response tendencies are larger for the less frequent/preferred primes (e.g. passives) than for more frequent/preferred primes (e.g. actives). So far, considering these first three features, the error-based implicit learning, inverse-frequency and surprisal accounts are good candidates. However, a fourth feature that the mechanism would have to be able to account for, is that syntactic priming effects manifest themselves differently in the response tendencies and the response latencies. In response latencies the effects are larger for the *more* frequent/preferred primes (e.g. actives). One possible suggestion is that (existing) theories could incorporate a competition mechanism as described in the previous section; other suggestions could be proposed and tested in future experiments. The fifth feature which our current findings shed light on is that response tendency effects are boosted by lexical overlap between the prime and target sentence, while the response latency benefit is not influenced by lexical overlap. A final piece of this puzzle is the time course of syntactic priming effects. Our experiments did not include a timing manipulation, but, while response tendency effects are found to be relatively long-lived, Wheeldon and Smith [18] have observed that response latency effects are short-lived [32], [38], [39]. To further shape the theories of syntactic processing, we believe that future studies should not focus exclusively on effects in response tendencies but also investigate effects in response latencies.

## Supporting Information

Appendix S1 List of the transitive verbs depicted in the stimuli.
(DOC)
