# Supplementary material for: A Paradox of Syntactic Priming: Why Response Tendencies Show Priming for Passives, and Response Latencies Show Priming for Actives
Source: PLoS One. 2011 Oct 11;6(10):e24209. doi: 10.1371/journal.pone.0024209 (PMC3191135; doi:10.1371/journal.pone.0024209)
Supplement: Appendix S1 — List of the transitive verbs depicted in the stimuli. (DOC) [file pone.0024209.s001.doc]

**Appendix S1**

List of the transitive verbs depicted in the stimuli.

| Transitive verbs in the Dutch infinitive | English translation |
| --- | --- |
| Used during Experiment 1 and Experiment 2 | |
| aankleden | to dress |
| achtervolgen | to follow |
| afdrogen | to dry |
| bangmaken | to scare |
| bedienen | to serve |
| bedreigen | to threaten |
| betalen | to pay |
| begroeten | to greet |
| duwen | to push |
| fotograferen | to photograph |
| interviewen | to interview |
| helpen | to help |
| knuffelen | to embrace |
| masseren | to massage |
| meetrekken | to pull |
| meten | to measure |
| optillen | to lift |
| overeindhelpen | to help getting up |
| omtrekken | to pull down |
| natmaken | to wet |
| neerschieten | to shoot down |
| schoppen | to kick |
| pesten | to tease |
| slaan | to hit |
| slepen | to drag |
| stoppen | to stop |
| tekenen | to draw |
| naroepen | to call to |
| troosten | to comfort |
| uitzwaaien | to wave goodbye |
| vastbinden | to tie |
| verzorgen | to look after |
| vinden | to find |
| voeren | to feed |
| wegsturen | to send away |
| wurgen | to strangle |
| Used additionally during Experiment 2 | |
| bekogelen | to pelt |
| kussen | to kiss |
| opmaken | to make up |
| straffen | to punish |
| vervoeren | to transport |
| Note: The left column lists the verbs in the Dutch infinitive form as they were presented before the picture and the right column lists the English translations | |
